# Vision Impairment in Dementia Care

**DOI:** 10.1093/geroni/igab046.325

**Published:** 2021-12-17

**Authors:** Heather Whitson

**Affiliations:** Duke University School of Medicine, Durham, North Carolina, United States

## Abstract

Epidemiological evidence indicates that 3-4% of community-dwelling adults over age 65 years old have functionally limiting deficits in both vision and cognition. The comorbidity prevalence is higher in older age strata and in long-term care. Seniors with co-occurrence of vision impairment and dementia have six times higher odds of disability and higher average annual Medicare fee for service costs ($13,655 [95% confidence interval: $9,931-$18,798], compared to peers with dementia alone ($8,867 [95% confidence interval: $7,360-10,683]) or neither condition ($4,518 [95% confidence interval: $4,360-$4,682]). This talk will review evidence that people with early dementia and vision problems can experience improved function through appropriately tailored vision rehabilitation interventions. The talk will provide recommendations for unbiased cognitive assessment in visually impaired people. The session will outline research opportunities regarding the question of whether preventing or treating vision impairment may improve cognitive trajectories and neuropsychiatric symptoms in people with dementia.

